# A simple prediction score system for malignant brain edema progression in large hemispheric infarction

**DOI:** 10.1371/journal.pone.0171425

**Published:** 2017-02-08

**Authors:** KwangWook Jo, Suhas S. Bajgur, Hoon Kim, Huimahn A. Choi, Pil-Woo Huh, Kiwon Lee

**Affiliations:** 1 Department of Neurosurgery, Bucheon St. Mary's Hospital, College of Medicine, The Catholic University of Korea, Seoul, Republic of Korea; 2 Department of Neurosurgery, School of Medicine, University of Texas, Houston, Texas, United States of America; 3 Department of Neurosurgery, Uijeongbu St. Mary's Hospital, College of Medicine, The Catholic University of Korea, Seoul, Republic of Korea; University of Brescia, ITALY

## Abstract

Malignant brain edema (MBE) due to hemispheric infarction can result in brain herniation, poor outcomes, and death; outcome may be improved if certain interventions, such as decompressive craniectomy, are performed early. We sought to generate a prediction score to easily identify those patients at high risk for MBE. 121 patients with large hemispheric infarction (LHI) (2011 to 2014) were included. Patients were divided into two groups: those who developed MBE and those who did not. Independent predictors of MBE were identified by logistic regression and a score was developed. Four factors were independently associated with MBE: baseline National Institutes of Health Stroke Scale (NIHSS) score (p = 0.048), Alberta Stroke Program Early Computed Tomography Score (ASPECTS) (p = 0.007), collateral score (CS) (p<0.001) and revascularization failure (p = 0.013). Points were assigned for each factor as follows: NIHSS ≤ 8 (= 0), 9–17 (= 1), ≥ 18 (= 2); ASPECTS≤ 7 (= 1), >8 (= 0); CS<2 (= 1), ≥2 (= 0); revascularization failure (= 1),success (= 0). The MBE Score (MBES) represents the sum of these individual points. Of 26 patients with a MBES of 0 to 1, none developed MBE. All patients with a MBES of 6 developed MBE. Both MBE development and functional outcomes were strongly associated with the MBES (p = 0.007 and 0.002, respectively). The MBE score is a simple reliable tool for the prediction of MBE.

## Introduction

Malignant large hemispheric infarction (LHI) constitutes approximately 10% of supratentorial acute ischemic stroke (AIS) and has historically been associated with high morbidity and mortality. Secondary brain injury is primarily caused by malignant brain edema (MBE) that can lead to irreversible tissue damage, inadequate cerebral blood flow, increased intracranial pressure (ICP), and brain herniation [1–3]. Early decompressive craniectomy (DC) has been demonstrated to be an effective treatment strategy to reduce morbidity and mortality in several randomized controlled studies [4–7], although the ideal candidate for DC and exact timing of DC continues to be debated. Being able to identify those patients at highest risk for MBE and therefore those who may particularly benefit from early DC is important. Previous studies have identified several risk factors for the development of MBE such as: initial National Institutes of Health Stroke Scale (NIHSS) score, Alberta Stroke Program Early CT Score (ASPECTS), collateral score (CS), clot burden score (CBS) and diffusion/perfusion parameters in magnetic resonance image (MRI) [8–12].That being said, no simple prediction score for the development of MBE has yet been developed. The aim of this study was to identify independent risk factors for the development of MBE in order to generate a simple, reliable prediction score based on strength of association of each independent risk factor.

## Subjects and methods

### Patients

Institutional review board approval of Bucheon St. Mary’s Hospitlal of The catholic university of Korea was obtained for all aspects of this study (HC13RISI0087). Demographic and clinical information, baseline laboratory values, and radiologic image information was analyzed retrospectively from the stroke database of a single academic institution from January 2011 through September 2014. The severity of the patients’ initial neurological deficits was assessed via NIHSS by an experienced neurosurgeon and neurologist in the emergency department. The inclusion criteria for our study were as follows: (1) adult patients (≥18 years) with clinical and computed tomography (CT) evidence of acute ischemic stroke with CT angiography (CTA) evidence of proximal MCA occlusion, with or without internal carotid artery (ICA) occlusion; (2) MRI confirmation of acute ischemic stroke via diffusion-weighted imaging (DWI); and (3) availability of follow-up CT/CTA. Exclusion criteria were: (1) concurrent hemorrhagic stroke; (2) concurrent infarction in remote vascular territories; and (3) comorbidities likely to influence both pre-morbid and long-term functional outcomes (previous disabling neurological disease, dementia, terminal illness).

### Image acquisition

All patients received urgent acute stroke imaging (non-contrast CT, CTA) in the emergency department, acquired using a standardized protocol with a 64 slice multidetector CT scanner. Axial maximum intensity projection (MIP) images were obtained with 5 mm slices [9]. The ASPECTS, CBS, and CS were independently adjudicated on the patient’s baseline scans by two experienced neurosurgeons (H.K. and K.W.J.), as previously described [7,9,13].MRI was performed on every patient to confirm AIS and determine the exact extent of infarction. Revascularization was determined via 1) peri-procedural angiography if the patient was eligible for intra-arterial therapy, and/or 2) a 24 hour post-stroke CTA, obtained in all patients. On follow-up CTA, revascularization was defined as the visualization of distal vessels in the distribution of the previously occluded MCA compared to initial CTA. A non-contrast head CT was also performed at 24 hours post-stroke in all patients to reassess brain edema. Additional imaging was then performed as needed by the clinical team.

### Treatment protocol

Intravenous tissue plasminogen activator (t-PA) was given to those patients within a 3- hour time window from symptom onset. As CTA evidence of proximal large vessel occlusion was an inclusion criterion in our study, all patients within a 6 hour time window then underwent diagnostic angiography. If a proximal vessel occlusion was confirmed on angiography, intra-arterial mechanical thrombectomy was attempted using the Solitaire FR Revascularization Device.

All patients were subsequently admitted to the intensive care unit and treated according to standard protocols. All patients meeting both radiologic criteria for MBE and neurologic deterioration (NIHSS increase by greater than 2 points and decrease in the level of consciousness to a score of 1 or greater on item 1A of the NIHSS) were considered for decompressive craniectomy within 72 hours from stroke ictus without any age cut-off. The decision to perform DC was made in consultation with the patient/patient’s family and full written informed consent was obtained.

### Definition of MBE cohort

The patients were divided into those who developed MBE versus those who did not. The MBE cohort was defined in accordance with previously published studies [14–16] as those patients with: 1) acute, complete MCA infarction with early parenchymal hypodensity of at least 50% of the MCA territory and signs of local brain swelling such as sulcal effacement and compression of the lateral ventricle; 2) midline shift of >5 mm at the septum pellucidum or pineal gland with obliteration of the basal cisterns; and 3) neurological deterioration consisting of a NIHSS increase by >2 points and decrease in the level of consciousness to a score of ≥ 1 on item 1A of the NIHSS.

### Clinical assessments

Early neurological deficits were assessed via the NIHSS by an attending neurosurgeon and intensivist [17]. Functional outcome at 90 days after symptom onset was evaluated by a rehabilitation attending using the Modified Rankin Scale (mRS) score. Favorable outcome was defined as a mRS of ≤2 and unfavorable outcome was defined as a mRS of ≥ 3 [18].

### Selecting the scoring variables

We pre-specified age, gender, NIHSS, ASPECTS, clot burden score (CBS), collateral score (CS), clot location and revascularization status as probable predictors of MBE. These are parameters that could be determined within few hours of admission. The NIHSS is a standard tool for clinical assessment in AIS and baseline NIHSS is a commonly used predictor of outcome [10,19]. The NIHSS score ranges from 0–42 with higher score suggesting poor outcome. Various cut-offs have been recommended to determine severity of stroke [9,10,19]. ASPECT score is another useful scoring scale for assessing severity of LHI using CT [20]. One point is subtracted from a total of 10 to indicate evidence of a focal hypodensity and/or loss of gray/white matter differentiation in each of the 10 ASPECTS regions [20,21]. A score of 10 is normal, while a score of 0 reflects diffuse ischemic involvement in the whole MCA territory. Lower ASPECT scores are associated with greater extent of ischemic lesion in the MCA territory [22]. Clot Burden Score determines the extent of the clot in proximal anterior circulation by location and is graded on a scale of 0–10, 2 points are subtracted for a thrombus present in each of the supraclinoid segment and 1 point is subtracted for thrombus present in each of the infraclinoid segment, a score of 0 indicates complete vessel occlusion [23]. Leptomeningeal collateral circulation is critical in maintaining blood flow to the ischemic regions to reduce ischemic injury [24,25]. Despite its importance, it has been difficult to simply and quantitatively measure the degree of collateral flow. The CS is a useful tool for assessing leptomeningeal collateral circulation on CTA [9]. The range of CS scores was 0 to 3. (0 is absence of collateral supply to the occluded MCA territory. 1 is collateral supply ≤50% but >0% of the occluded MCA territory, 2 is collateral supply <100% but >50% of the occluded MCA territory, 3 is normal or greater compared to the normal contralateral hemisphere). Early recanalization may help to prevent extent of ischemic core and survive penumbra area and lead to improved clinical outcomes. Recent studies have shown rapid endovascular recanalization has been shown to improve clinical outcomes [26–30].

### Statistical analyses

Overall number (%), mean and standard deviation (SD), median and interquartile range (IQR) are reported as appropriate. Age, NIHSS, ASPECTS, CBS, and CS were considered continuous variables, while gender, lesion location, and revascularization status as categorical variables. Student’s *t*-test was used to analyze continuous variables, and *χ*^*2*^ or Fisher’s exact testto analyze categorical variables. Mann-Whitney *U-*test was used to compare medians between groups. Univariate analysis was done using logistic regression for continuous variables and *χ*^*2*^ or Fisher’s exact test for categorical variables. Variables with probability <0.2 from the univariate analysis were then entered into multivariate logistic regression model using forced entry method using only 3 variables at a time, thus meeting the recommendation of 10–15 events per predictor [31]. To assess the calibration of the model we used the Hosmer-Lemeshow *χ*^*2*^ test (*P>0*.*05*) [32]. The variance of the prediction model is described using Nagelkerke *R*^*2*^. Internal validation was performed using regular bootstrap sampling with 1000 repetitions [33,34]. The continuous variables were then categorized based on medians or tertiles and points were assigned to each category based on strength of association with MBE. The points were then summated to obtain the final prediction score. The area under the receiver operating characteristics (ROC) curve was used to assess the discrimination of the prediction score [35]. The threshold for statistical significance was set at *p*< 0.05. All statistical analyses were performed using the Statistical Package for the Social Sciences software version 22 (SPSS, Chicago, IL, USA).

## Results

### Baseline patient demographics

One hundred twenty-one patients with LHI hospitalized during January 2011 to September 2014were analyzed. Mean patient age was 68 years (±13.7), with 62 males (51%). The right hemisphere was infarcted in 64 patients (53%). 52 patients (43%) had evidence of carotid T-occlusion. The median (IQR) baseline NIHSS score was 12(8–22). The median (IQR) ASPECTS, CBS, and CS as assessed on initial CT/CTA were 7 (4–7), 6 (5–9), and 2 (0–2), respectively. Recanalization as evident on 24 hour follow-up CTA was achieved in 57(47%) patients. 36 patients (29.8%) developed MBE. The median (IQR) 90 day mRS was 4 (1–5). Demographic data is summarized in Table 1.

**Table 1:** Baseline Patient Characteristics.

		N = 121
**Demographic**		
	Age, mean (SD)	68 (13.7)
	Female, n (%)	59 (49)
	NIHSS, median (IQR)	12 (8–20)
**Medical History**		
	DM type II, n (%)	19 (15.7)
	Hypertension, n (%)	73 (60.3)
	Hyperlipidemia, n (%)	19 (15.7)
	Atrial Fibrillation, n (%)	33 (27)
**Lesion Location**		
	MCA	69 (57)
	T-Occlusion	52 (43)
**Lesion Side**		
	Right	64 (53)
	Left	57 (47)
**Radiographic Scores, median (IQR)**		
	ASPECTS Score	7 (5–9)
	Clot Burden Score	6 (4–7)
	Collateral Score	2 (1–2)
**Lab Values, mean (SD)**		
	Glucose	130 (44.3)
	Hemoglobin	13.6 (1.74)
	WBC x 10^3^	9.88 (4.51)
	Platelet Count x 10^3^	222.5 (70)
	CRP	4.45 (8)
**Treatment & Outcome**		
	Revascularization Achieved, n (%)	57 (47)
	90 day mRS, median (IQR)	4 (1–5)

SD, Standard Deviation; IQR, Interquartile Range; NIHSS, National Institute of Stroke Scale; DM, Diabetes Mellitus; MCA, Middle Cerebral Artery; ICA, Internal Carotid Artery; ASPECTS, Alberta Stroke Program Early CT Score; mRS, modified Rankin Score.

### Developing the MBE score (MBES)

**Fig 1:**
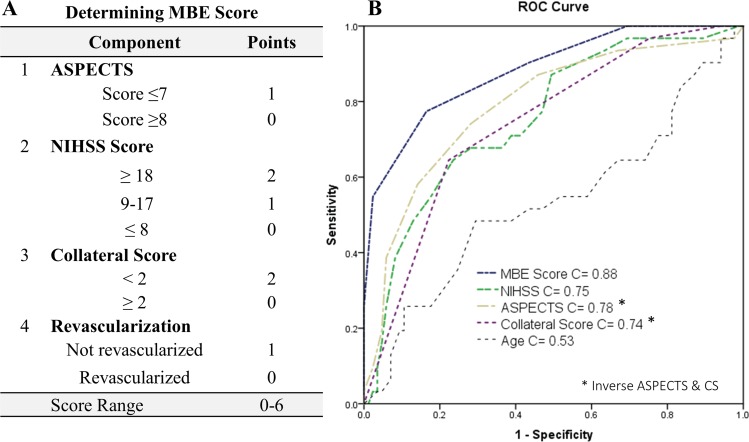
(A) MBE Scoring Components and Scores. (B) Receiver operator characteristics curve showing MBES in comparison with other clinical scores (NIHSS, ASPECTS, CS) that predict MBE. Age is shown for reference only.

Seven of the pre-specified variables that had a p-value of >0.2 (Table 2) were entered into a logistic regression model with three variables at a time, two of the continuous (ASPECTS, NIHSS, CBS, CS) and one of binary predictor variable (gender, location of clot, revascularization status) (S1 Table). We used this hypothesis driven approach to address the relatively small number of outcome events in our study (N = 36) and to avoid overfitting the logistic regression model [31,36]. Four characteristic that were found to be significant across various models were then entered into a final logistic regression model to confirm if they independently predicted MBE (Table 3). An MBE risk stratification score was developed with points assigned based on strength of association. We divided the baseline NIHSS score into 3 subgroups based on study population tertiles: NIHSS ≤ 8, 9 to 17, and ≥ 18 and points were assigned 0, 1 and 2 respectively. We divided our population by tertiles to account for the score spread (0–42) and yet provide weight age comparable to other stronger predictor like CS. ASPECTS and CS were dichotomized based on medians. ASPECTS ≤ 7 received 1 point and zero if more than 7. CS was found to be the strongest independent predictor and therefore a score of <2 earned 2 points. One point was assigned if there was failure to revascularize. Individual points were then summated to calculate the total MBE score (MBES), with a range from 0 to 6 (Fig 1A). Each of the categorized variables was strongly associated with MBE (Table 4).

**Table 2:** Univariate Analysis of Patient Characteristics.

		No MBE N = 85	MBE N = 36	*P* ^a^
**Age, mean ±SD**		67.7 (13)	69.5 (14)	0.43
**Female, n (%)**		33 (39)	26 (72)	0.001
**Scores, median (IQR)**				
	NIHSS	10 (7–17)	20 (11–22)	<0.001
	ASPECTS	8 (6–9)	5 (4–6)	<0.001
	Clot Burden Score	6 (4–7)	3 (1–6)	<0.001
	Collateral Score	2 (2–3)	1 (1–2)	<0.001
**Site of Occlusion, n (%)**				
	T-occlusion	28 (33)	24 (67)	0.001
**Not Revascularized, n (%)**		36 (42)	28 (78)	0.001

^a^ Logistic regression for continuous variables.

**Table 3:** Predictors of MBE.

			95% Confidence Interval	
	*P*	Odds Ratio	Lower	Upper
**NIHSS**	.048	1.092	1.001	1.193
**ASPECTS**	.007	.666	.496	.895
**Collateral Score**	.000	.165	.064	.426
**Revascularization (No)**	.013	4.371	1.369	13.956

**Table 4:** Association of categorized predictors with MBE.

Categorized Variables N (%)		No MBE N = 85	MBE N = 36	Odds Ratio (95% CI)	*P*
**NIHSS**^a^					
	≤8	31 (36)	2 (5.6)		
	9 to 17	34 (40)	11 (30)	5 (1.02–24.4)	0.046
	≥18	20 (24)	23 (64)	17.8 (3.7–84)	<0.001
**ASPECTS**^b^					
	≤7	39 (46)	32 (89)	9.4 (3.0–29.0)	<0.001
**Collateral Score**^b^					
	<2	19 (22)	25 (69)	7.9 (3.3–18.9)	<0.001
**Revascularization (No)**					
		36 (42)	28 (78)	4.7 (1.9–11.6)	0.001

^a^ NIHSS categories are based on study population tertiles.

^b^ ASPECTS, CBS and CS categories are based on study population median.

### MBES validation

**Fig 2:**
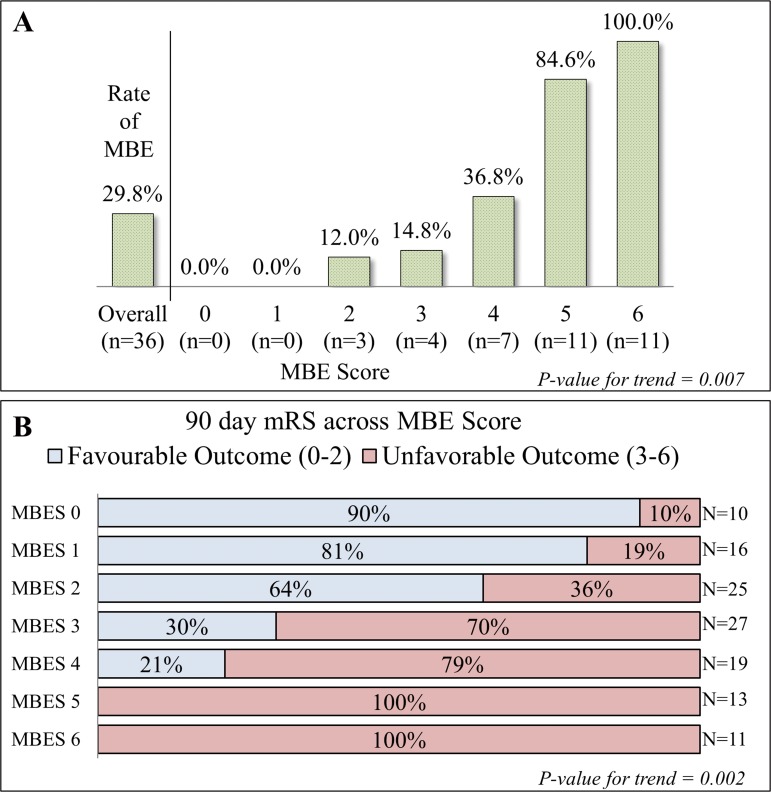
(A) Rate of MBE across MBESs. (B) 90 day mRS score distribution across MBESs.

The area under the ROC curve was 0.88 for prediction of MBE using MBES (Fig 1B). For the logistic regression model Hosmer-Lemeshow *χ*^*2*^and p-value were 3.87 and 0.86 respectively and Nagelkerke *R*^*2*^ was 0.59. A score of 5 or more had a sensitivity of 61% and a specificity of 97.6% for detection of MBE. The positive predictive value and negative predictive values were 91.7% and 85.6% respectively. The bootstrap corrected p-values were significant for all four predictor variables. Percentage of patients who developed MBE as stratified by MBES is presented in Fig 2A. None of the patients with MBES of 0 and 1 developed MBE, whereas all patients with a score of 6 developed MBE.

### MBES and outcome

The MBES was strongly associated with 90 day functional outcomes (p = 0.002). Patients with an MBES of 0–2 were more likely to have favorable 90 day mRS as compared to patients with a MBES of 3–6 (OR: 14.1, CI: 5.8–34.2, p<0.001). All patients with a MBES of 5 or 6 had unfavorable outcomes at 90 days (Fig 2B). Regarding mortality, the overall mortality was 9.1% (11 patients). 8 patients (73%) with a MBES of 6 died and remaining 3 survived with severe disability (mRS 5).

## Discussion

Although early DC has been shown to be effective in reducing mortality and morbidity in patients with MBE [4–6,37], early DC before the onset of neurologic deterioration still is not commonly performed because of uncertainty of edema progression and surgery-related complications [8,9,38]. Any score that predicts the risk of MBE development would be a clinically useful tool for decision making at bedside especially to ascertain the timing of DC [8–12]. The objective of this study was to develop a simple, reliable prediction score using the known independent risk factors.

### Understanding MBES

Clinical scoring systems provide an objective method of assessing disease severity and guides decision making. MBES is one such scoring system that we hope serves this purpose. We found four characteristics that independently predict MBE: baseline NIHSS, ASPECTS, CS, revascularization status. NIHSS has been in use for over a decade and its clinical utility is well described in the literature. Numerous studies have described NIHSS as a predictor of outcome. In this study we have shown that NIHSS also predicts MBE, and can be used in conjunction with other predictors to determine risk of MBE. Similar to our previous experience, an NIHSS≥18 was strongly associated with MBE [9].The ASPECTS is a reliable and reproducible way of quantifying the extent of parenchymal tissue damage after LHI [21]. The original ASPECTS study as well as a numerous other studies have clearly identified poor outcome in patients with score ≤7 [22]. Our study further indicates that patients with ASPECTS ≤ 7 tend to develop MBE. Presence of good collateral circulation sustains brain viability to arterial occlusion [24] on the other hand poor leptomeningeal collaterals as indicated by low CS of 0 and 1 can be ominous of continued tissue damage in presence of an ischemic insult [13]. In our study a CS of <2 strongly predicted MBE and is an important component of this scoring system.

DWI volume and Time to Peak (TTP) >+4s volumes were also powerful predictive factors. Assessment of perfusion by collateral flow and ischemic area volume is important. Although ASPECT and CS represent the extent of ischemic area and collateral flow, it does not show the perfusion state and ischemic core size. Thomalla GJ et al suggested that large DWI volume (>82 mL) and TTP >+4s volume (>162 mL) are predictive factors [39]. We acknowledge the importance of infarct volume measured by DWI. However, we chose not to include this parameter because we believe that a prediction score should be simple and feasible. In general, MRI is not the first choice for patients with suspected ischemic stroke in emergency settings because it is time consuming, and it is not always possible to conduct an MRI study in every hospital. Volume calculation and TTP measurements are not simple and need specialized programs and are not feasible especially in emergent situations. In addition, emergent perfusion MRI is often logistically difficult to perform in LHI patients.

Age is generally considered an important predictor of outcome. However, older age is also closely tied to brain atrophy and the amount of “room” available for brain edema to occur. In this study, we did not find an association between age and MBE. This may be due to a high number of older patients in our study population (54% older than 68 years). Further studies are warranted to understand the effect of age on MBE.

### Decision making and MBES

Our results may provide some clues in making acute treatment decisions in patients with LHI. Patients with an MBES of 0–1 had no MBE and good outcome after medical treatment (90%, 81.3% respectively). In this group, initial medical treatment may be more beneficial. Patient with a MBES of 2 showed a slight increase in the risk of MBE (12.0%) and poor outcome (36.0%). In our study, 3 patients had a MBES of 2, all patients underwent medical treatment none died and they had a relatively high rate of good outcome (64%). In this group, close observation for MBE is needed to reduce morbidity. Patients with a MBES of 3–4 showed high risk of poor outcome (70.4%, 78.9%) although they showed relatively low rate of MBE (14.8%, 36.8%). In patients with a MBES of 4, 5 patients (26.3%) underwent DC within 72hrs. Two of them who underwent DC *after 48hrs* of ictus died however all patient who underwent DC *within 48hrs* survived and one among them showed good outcome (mRS 2) after DC. In this group, early DC (<48hrs) may be helpful to reduce mortality and morbidity. In the group with MBES of 5–6, all patients showed poor outcome regardless of surgical and medical treatment. In this group, DC should be considered only as a lifesaving measure because most patients remain severe disability. Quality of life and economic burden of long-term care should be taken into consideration in this group of patients. While, treatment decisions are currently made on a case-by-case basis according to the patient's clinical status and wishes and the expectations of their family members. MBES could be useful in surgical decision making and its role remains to be established in future studies.

### Study limitations

This study has several limitations. First, this is a retrospective study, conducted with relatively a small number of patients of a single center. Second the number of outcome events (MBE) was small for multivariate analysis and therefore we had to be cautious about not over fitting the model, a validation of this model in larger study population would address this issue. Third this prediction score is based on a single-center experience and therefore needs to be externally validated before wider deployment. And finally with regards to DC, MBES is reliable for prediction of MBE and outcome according to standard treatment. However, these results may not be applicable when prophylactic DC is done (before any neurological deterioration). Our patients underwent DC after confirmation of MBE by neurologic examination and CT. MBES provides no information about the accurate timing of life-saving procedure like DC. A prospective trial is warranted to explore the relationship between MBES and timing of surgery.

True natural history of MBES 5–6 is unclear. If people withdraw care or withhold DC in this group because they believe everyone would have a poor outcome, this will be a “self-fulfilling prophecy”. It is particularly dangerous in younger patients who are likely have a great chance of survival with less disability. Further studies are needed to accurately assess the outcome of MBES 5–6, even in cases where they receive aggressive care, to ensure that their outcomes are truly poor.

## Conclusion

This report of MBES is a preliminary and is the first simple grading scale for prediction of MBE progression and outcome. The MBES needs further research and validation to be used decision making in early aggressive treatment such as DC in patients with LHI. Also this decision making should include the patient's overall clinical status, expected quality of life, and wishes of patients and family.

## Supporting information

S1 Table Predictors of MBE (3 predictors at a time). (DOCX)
