# Supplementary material for: A simple prediction score system for malignant brain edema progression in large hemispheric infarction
Source: PLoS One. 2017 Feb 8;12(2):e0171425. doi: 10.1371/journal.pone.0171425 (PMC5298259; doi:10.1371/journal.pone.0171425)
Supplement: S1 Table — (DOCX) [file pone.0171425.s001.docx]

**Supporting Information**

**S1 Table. Predictors of MBE (3 predictors at a time)**

|  |  |  |  | **95 % Confidence Interval** | |
| --- | --- | --- | --- | --- | --- |
|  | **Variable Groups** | ***P*** | **Odds Ratio** | **Lower** | **Upper** |
| 1 |  |  |  |  |  |
|  | NIHSS | **.031** | 1.086 | 1.008 | 1.171 |
|  | ASPECTS | **.001** | .633 | .488 | .821 |
|  | Gender(Female) | .073 | 2.435 | .920 | 6.442 |
| 2 |  |  |  |  |  |
|  | NIHSS | **.036** | 1.085 | 1.005 | 1.170 |
|  | ASPECTS | **.000** | .629 | .486 | .814 |
|  | Site of Occlusion (Carotid-T) | .109 | 2.187 | .839 | 5.699 |
| 3 |  |  |  |  |  |
|  | NIHSS | **.036** | 1.084 | 1.005 | 1.170 |
|  | ASPECTS | **.000** | .629 | .487 | .812 |
|  | Revascularization (No) | **.021** | 3.303 | 1.200 | 9.096 |
| 4 |  |  |  |  |  |
|  | NIHSS | **.004** | 1.110 | 1.033 | 1.192 |
|  | Clot Burden Score | **.003** | .745 | .612 | .908 |
|  | Gender(Female) | **.018** | 3.142 | 1.218 | 8.107 |
| 5 |  |  |  |  |  |
|  | NIHSS | **.006** | 1.106 | 1.029 | 1.189 |
|  | Clot Burden Score | **.008** | .764 | .627 | .932 |
|  | Site of Occlusion (Carotid-T) | .087 | 2.269 | .887 | 5.803 |
| 6 |  |  |  |  |  |
|  | NIHSS | **.007** | 1.105 | 1.028 | 1.189 |
|  | Clot Burden Score | **.005** | .754 | .619 | .919 |
|  | Revascularization (No) | **.013** | 3.469 | 1.296 | 9.286 |
| 7 |  |  |  |  |  |
|  | NIHSS | **.002** | 1.134 | 1.046 | 1.228 |
|  | Collateral Score | **.000** | .177 | .078 | .399 |
|  | Gender(Female) | **.023** | 3.325 | 1.184 | 9.335 |
| 8 |  |  |  |  |  |
|  | NIHSS | **.002** | 1.139 | 1.050 | 1.236 |
|  | Collateral Score | **.000** | .180 | .079 | .408 |
|  | Site of Occlusion (Carotid-T) | .110 | 2.256 | .833 | 6.109 |
| 9 |  |  |  |  |  |
|  | NIHSS | **.003** | 1.130 | 1.042 | 1.226 |
|  | Collateral Score | **.000** | .139 | .055 | .352 |
|  | Revascularization (No) | **.003** | 5.678 | 1.829 | 17.626 |
| 10 |  |  |  |  |  |
|  | Clot Burden Score | .145 | .844 | .672 | 1.060 |
|  | Collateral Score | **.000** | .232 | .103 | .522 |
|  | Gender(Female) | **.007** | 3.902 | 1.458 | 10.441 |
| 11 |  |  |  |  |  |
|  | Clot Burden Score | .174 | .859 | .690 | 1.070 |
|  | Collateral Score | **.001** | .240 | .107 | .540 |
|  | Site of Occlusion (Carotid-T) | **.038** | 2.750 | 1.056 | 7.162 |
| 12 |  |  |  |  |  |
|  | Clot Burden Score | .282 | .882 | .701 | 1.109 |
|  | Collateral Score | **.000** | .170 | .067 | .433 |
|  | Revascularization (No) | **.001** | 6.296 | 2.112 | 18.773 |
| 13 |  |  |  |  |  |
|  | ASPECTS | **.000** | .606 | .458 | .801 |
|  | Collateral Score | **.000** | .206 | .090 | .472 |
|  | Gender(Female) | .078 | 2.599 | .897 | 7.530 |
| 14 |  |  |  |  |  |
|  | ASPECTS | **.000** | .596 | .452 | .787 |
|  | Collateral Score | **.000** | .218 | .096 | .495 |
|  | Site of Occlusion (Carotid-T) | .246 | 1.857 | .653 | 5.282 |
| 15 |  |  |  |  |  |
|  | ASPECTS | **.001** | .611 | .463 | .808 |
|  | Collateral Score | **.000** | .172 | .069 | .430 |
|  | Revascularization (No) | **.007** | 4.710 | 1.517 | 14.621 |
| 16 |  |  |  |  |  |
|  | ASPECTS | **.000** | .627 | .483 | .814 |
|  | Clot Burden Score | **.022** | .787 | .641 | .967 |
|  | Gender(Female) | .076 | 2.413 | .912 | 6.381 |
| 17 |  |  |  |  |  |
|  | ASPECTS | **.000** | .619 | .477 | .802 |
|  | Clot Burden Score | **.036** | .805 | .657 | .986 |
|  | Site of Occlusion (Carotid-T) | .158 | 2.016 | .762 | 5.334 |
| 18 |  |  |  |  |  |
|  | ASPECTS | **.000** | .625 | .484 | .807 |
|  | Clot Burden Score | **.026** | .791 | .644 | .972 |
|  | Revascularization (No) | **.022** | 3.284 | 1.191 | 9.055 |
